# Pancreaticopleural fistula in a Thai boy with *SPINK1* c.101A>G substitution variant-related chronic pancreatitis: a case report and literature review

**DOI:** 10.2478/abm-2022-0012

**Published:** 2022-04-29

**Authors:** Chomanad Chittchang, Nisa Netinatsunton, Supika Kritsaneepaiboon

**Affiliations:** Department of Radiology, Faculty of Medicine, Prince of Songkla University, Hat Yai, Songkhla 90110, Thailand; Department of Radiology, Queen Sirikit National Institute of Child Health, Ratchathewee, Bangkok 10400, Thailand; Nanthana-Kriangkrai Chotiwattanaphan (NKC) Institute of Gastroenterology and Hepatology, Songklanagarind Hospital, Department of Internal Medicine, Prince of Songkla University, Hat Yai, Songkhla 90110, Thailand

**Keywords:** cholangiopancreatography, magnetic resonance, fistula, hereditary pancreatitis, pancreatitis, chronic, pleural effusion

## Abstract

**Background:**

Chronic pancreatitis is the most common etiology of pancreaticopleural fistula (PPF) in children, and underlying genetic variations are now widely known, accounting for most chronic pediatric pancreatitis.

**Case report:**

We describe a case of previously undetected chronic pancreatitis and PPF with a *SPINK1* variation in a 10-year-old Thai boy who presented with massive left pleural effusion. Magnetic resonance cholangiopancreatography (MRCP) revealed disruption of the pancreatic duct, which was communicating with a large pancreatic pseudocyst with mediastinal extension. The patient subsequently underwent endoscopic intervention with improved clinical symptoms. We also reviewed the imaging findings of 12 other reported cases of pediatric PPF.

**Conclusions:**

Massive pleural effusion due to PPF can be an atypical manifestation in children with chronic pancreatitis. MRCP is the preferable imaging study for PPF due to the production of highly detailed images of pancreatic duct disruptions and anatomy, and the imaging is helpful to guide for appropriate treatment. Tests for genetic variation are also recommended in a child with chronic pancreatitis.

Pancreaticopleural fistula (PPF) is a rare complication of pancreatitis, especially chronic pancreatitis, with an incidence of about 0.4% in adult patients with chronic pancreatitis [1]. The PPF is even less commonly found in children because pediatric chronic pancreatitis is uncommon [1, 2]. The incidence of PPF in children has not been reported, but the fistula can cause significant pulmonary symptoms. However, the abnormality is often misdiagnosed, leading to prolonged hospitalization. Genetic variations are now widely known as a significant cause of chronic pediatric pancreatitis [2]. The most frequently detected variations in patients with European ancestry are in the genes for protease serine 1 (*PRSS1*; OMIM 276000), cystic fibrosis transmembrane conductance regulator (*CFTR*; OMIM 602421), chymotrypsin C (*CTRC*, OMIM 601405), and serine protease inhibitor Kazal type 1 (*SPINK1*; Gene ID: 6690) [3]. The *SPINK1* c.101A>G substitution variant (OMIM 167790) is most frequently detected in patients with European ancestry, while the *SPINK1* c.194 + 2T>C substitution variant has often been reported in patients with Asian ancestry [3]. Imaging modalities can help to establish a diagnosis of pancreatitis, to define the severity and complication of pancreatitis, and to guide for proper management. Although computed tomography (CT) provides a high sensitivity for detecting pancreatitis, it is limited in evaluating the ductal anatomy, and because of exposure of children to radiation [4,5,6]. Magnetic resonance cholangiopancreatography (MRCP) improves noninvasive visualization of the pancreatic ductal anatomy while avoiding radiation exposure [5]. MRCP also helps to demonstrate pancreatic duct abnormalities, including dilatation, irregularities, or strictures, and detect PPF in chronic pancreatitis [7].

Herein, we present the case of a Thai boy with *SPINK1* c.101A>G substitution variant-related chronic pancreatitis who developed PPF with a pancreatic cyst and large mediastinal extension. The imaging findings to diagnose PPF and management in this patient are reviewed and discussed. We also reviewed and analyzed the imaging findings of 12 other reported cases of PPF in children [1, 2, 8,9,10,11,12,13,14,15,16].

## Case report

**Figure 1:**
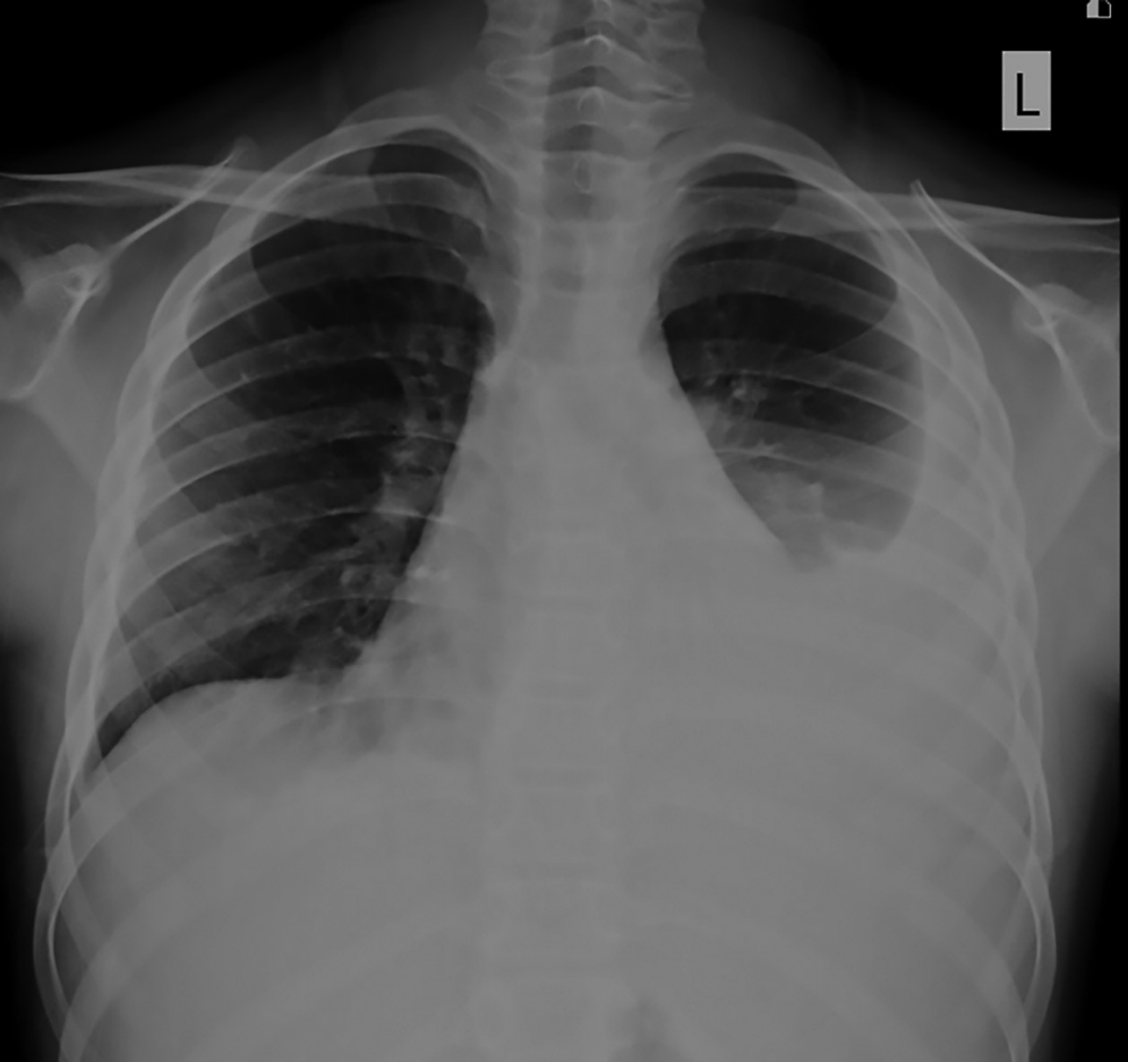
Chest radiograph in our patient showing massive left pleural effusion. The visualized upper abdomen appears unremarkable.

**Figure 2:**
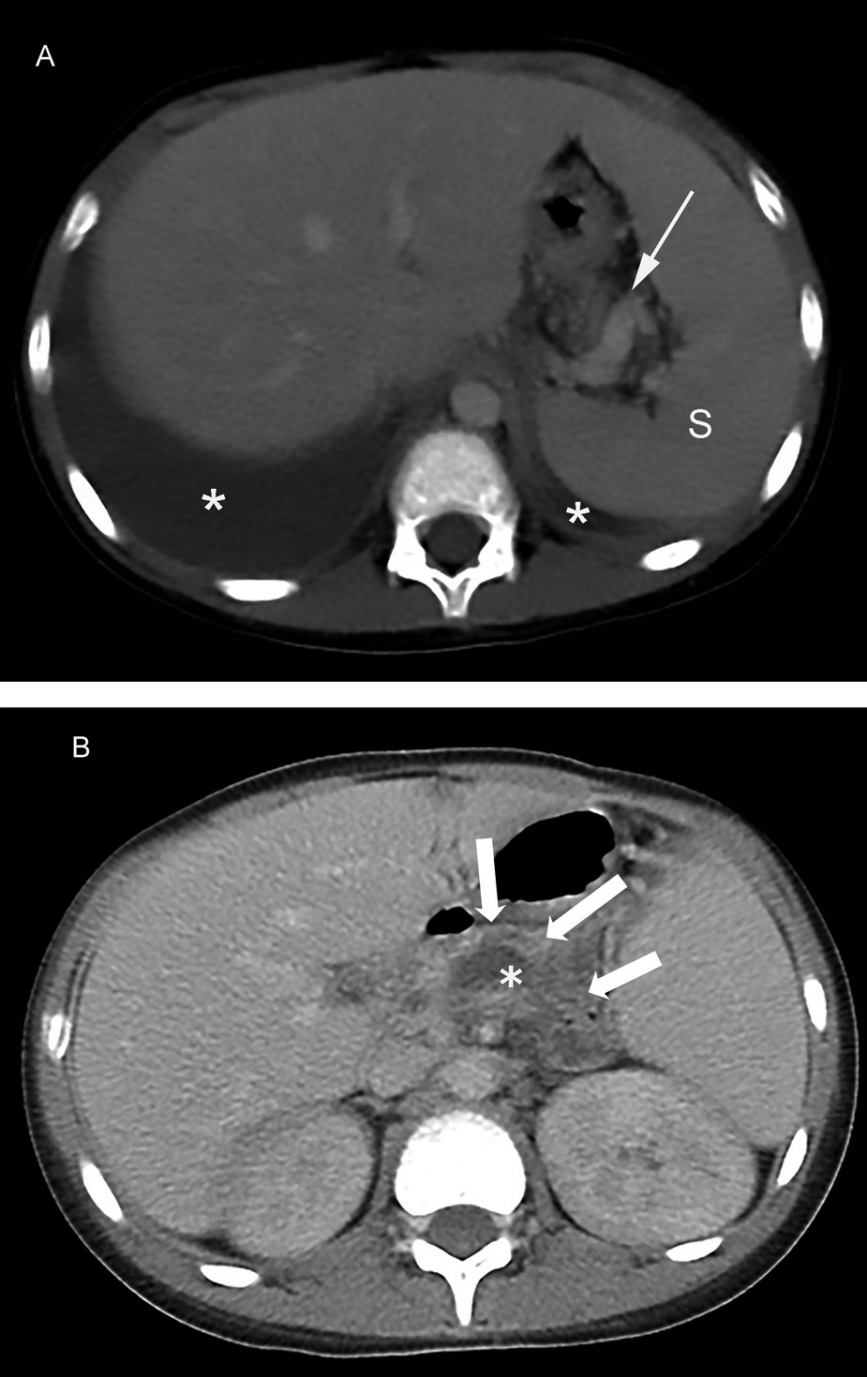
(**A**) An axial CT image in venous phase showing bilateral pleural effusions (asterisks). Splenomegaly (S) with splenic vein varices (arrow) is seen, indicating portal hypertension. However, no definite cause of portal hypertension was identified in this case. (**B**) The included upper abdominal part of the chest CT showing diffuse pancreatic atrophy and prominent pancreatic duct (arrows), consistent with chronic pancreatitis. A 2-cm-sized pseudocyst (asterisk) was observed, abutting the posterior aspect of the pancreatic body. No fistulous tract to the mediastinum is evident. CT, computed tomography.

A 10-year-old boy presented at a community hospital due to intermittent epigastric pain and weight loss of 8 kg in a month. An initial chest radiograph showed massive left pleural effusion (**Figure 1**). He underwent thoracic drainage. Pleural fluid analysis showed exudative profile, and was positive for anti-nuclear antibodies (ANA). He was treated with intravenous antibiotics and retained chest tube drainage for 6 days, the left pleural effusion had decreased. However, 4 days later, he complained of chest tightness and dyspnea, and chest radiography showed massive right pleural effusion. He was again treated with right chest tube drainage. Contrast-enhanced chest CT revealed bilateral pleural effusion (**Figure 2A**). Therefore, the patient was referred to Songklanagarind Hospital, a tertiary care, and teaching hospital of the Faculty of Medicine, Prince of Songkla University, Hat Yai, to determine the cause of pleural effusion. We obtained written informed consent in writing from the patient's parent to publish the present case report and associated images, and the CARE reporting guidelines were used when writing the report [17]. The patient information for this case was anonymized as far as possible to avoid identification of the patient or their family.

**Figure 3:**
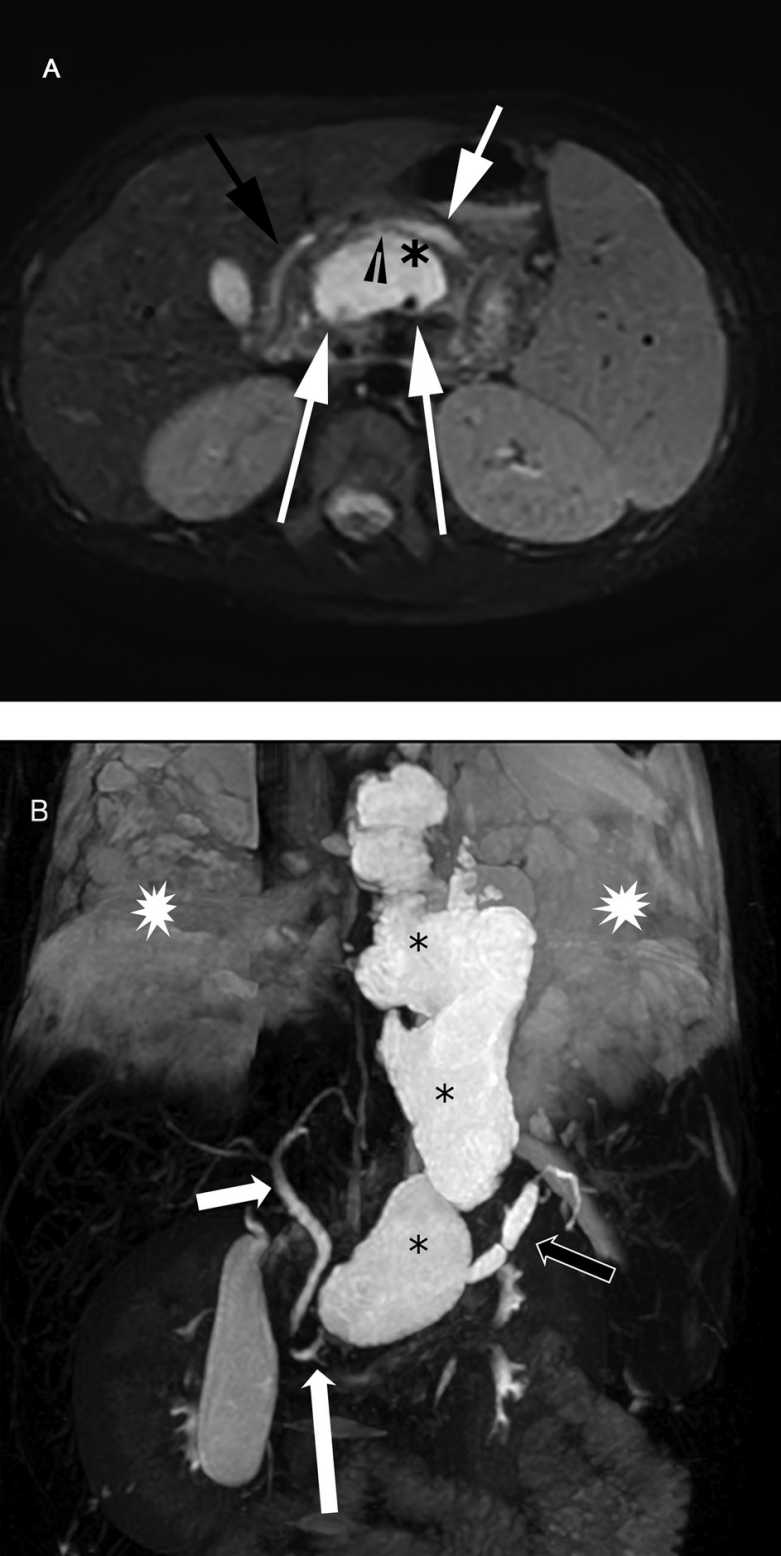
(**A**) An axial T2-weighted image with fat suppression showing thinning of the pancreatic parenchyma with a dilated upstream pancreatic duct (short white arrows), consistent with chronic pancreatitis. Disruption of the posterior aspect of the main (arrow head) can be seen, about 2 mm wide, connected with the pseudocyst (asterisk). A few stones (long white arrows) are observed within the pseudocyst. The downstream pancreatic duct (dark arrow) is narrower. (**B**) A 3-dimensional maximum intensity projection (MIP) image of MRCP showing a large pancreatic pseudocyst (asterisks) arising from the pancreatic genu-body junction and extending upward to the mediastinum. Disproportion of the non dilated downstream pancreatic duct (long white arrow) and the dilated upstream pancreatic duct (dark arrow) indicates pancreatic duct discontinuation. Massive bilateral pleural effusions (stars) can also be seen. The common bile duct (short white arrow) is not dilated. MIP, maximum intensity projection; MRCP, magnetic resonance cholangiopancreatography.

**Figure 4:**
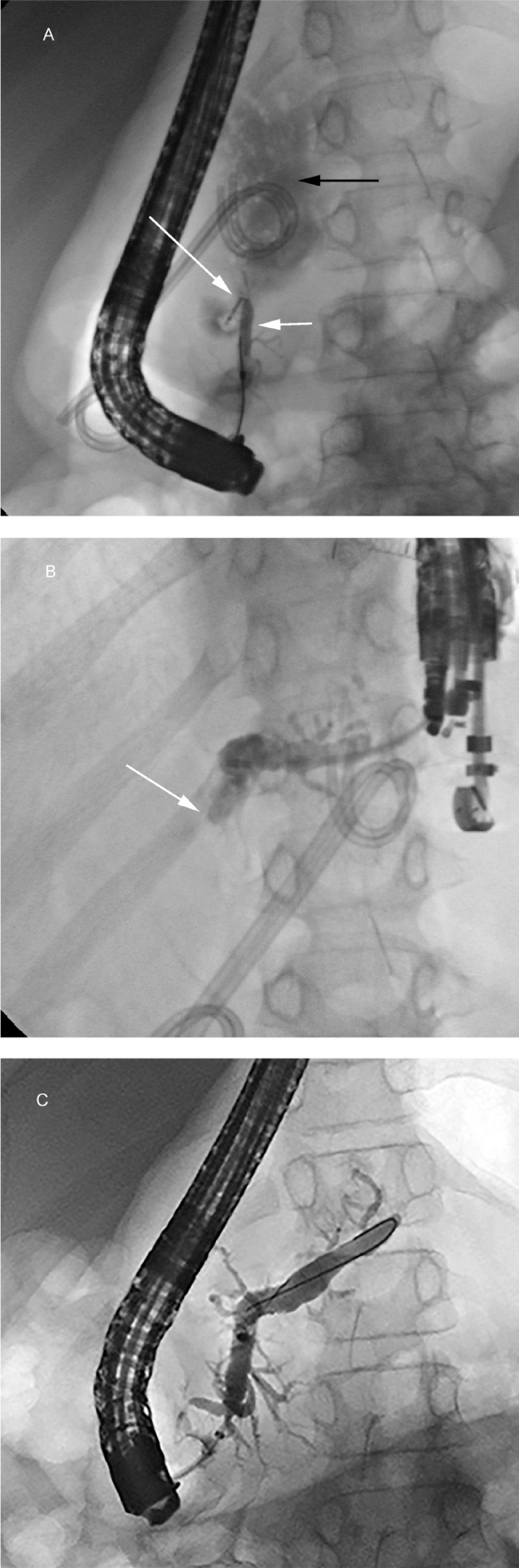
(**A**) ERCP showing opacification of contrast in the proximal pancreatic duct (short white arrow). Contrast leakage can be seen from the pancreatic duct at the genu-body junction of the pancreas (long white arrow) into the pseudocyst (dark arrow). No opacification of contrast in the distal part of pancreatic duct is evident. These findings are suggestive of complete disruption of the pancreatic duct (**B**) EUS-guided pancreatography showing opacification of contrast in the dilated upstream pancreatic duct only to the genu-body part of the pancreatic duct (arrow), with no further contrast filling in the more proximal pancreatic duct. These findings confirmed a diagnosis of disconnected pancreatic duct syndrome. (**C**) ERCP performed after 4 months with a retained pancreatic duct stent showing complete anatomical restoration of the pancreatic duct, representing successful endoscopic treatment. ERCP, endoscopic retrograde cholangiopancreatography; EUS, endoscopic ultrasound.

**Figure 5:**
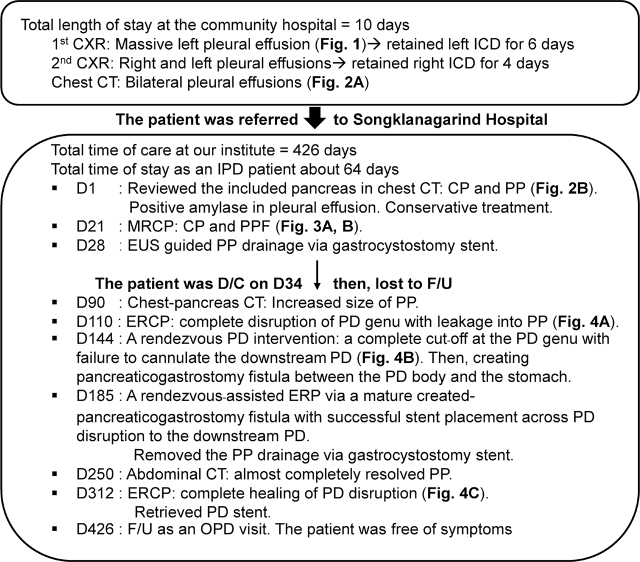
Summary of the episode of care in our case. CXR, chest radiograph; ICD, intercostal chest drain; CT, computed tomography; IPD, in-patient department; CP, chronic pancreatitis; PP, pancreatic pseudocyst; MRCP, magnetic resonance cholangiopancreatography; EUS, endoscopic ultrasound; D, day; D/C, discharged; F/U, follow up; ERCP, endoscopic retrograde cholangiopancreatography; PD, pancreatic duct; ERP, endoscopic retrograde pancreatography; OPD, out-patient department.

The patient's height was 145 cm and weight was 35 kg. He had no known underlying disease and no family history of pancreatitis. Physical examination revealed decreased breath sound on the right hemithorax. He had elevated serum amylase of 879 U/L and lipase of 817 U/L. Fasting lipids, blood glucose, and calcium levels were within normal range. The pleural fluid analysis revealed an elevated amylase level of 4079 U/L, but negative for ANA. Polymerase chain reaction (PCR) testing for tuberculosis, and bacterial cultures were negative for pathogenic organisms. Serology for autoimmune and connective tissue disease including anti-ds DNA, anti-cardiolipin, anti-B2-glycoprotein, and anti-Sm (Sm, small nuclear ribonucleoproteins autoantibodies) was all negative. The chest CT from the community hospital was reviewed and we found features of chronic pancreatitis including irregular pancreatic duct dilatation with diffuse atrophic pancreatic parenchyma, and a small pancreatic pseudocyst (**Figure 2B**). A diagnosis of pancreatitis was established and the patient was conservatively treated with fasting, parenteral nutrition, antibiotics, and octreotide for 10 days. The thoracic drainage was eventually removed. Subsequent MRCP performed on day 21 revealed evidence of chronic pancreatitis seen as thinning of the pancreatic parenchyma with irregular dilated upstream pancreatic duct and a focal disruption of the duct at the pancreatic genu-body connecting to a large pancreatic pseudocyst which extended upward to the mediastinum (**Figure 3A, B**). Hence, we diagnosed PPF complicated by chronic pancreatitis. Then endoscopic ultrasound (EUS)-guided gastrocystostomy pseudocyst drainage was successfully performed by placing two 7-Fr, 5-cm double pigtail stents between the body of the stomach and the pancreatic pseudocyst. Subsequently, the patient was discharged from the hospital and scheduled for a followed-up out-patient appointment at 1 month later. However, he was lost to follow-up at his out-patient appointment. A chest-abdominal CT performed on day 90 showed interval increase in the size of the pancreatic pseudocyst. Three weeks later, the patient underwent an endoscopic retrograde cholangiopancreatography (ERCP), which confirmed the MRCP findings of ductal disruption with a disconnected pancreatic duct genu communicating with the pseudocyst (**Figure 4A**). However, the endoscopic interventionist was unable to pass the ERCP wire distally beyond the disrupted pancreatic duct. A month later, a pancreatic sphincterotomy with rendezvous pancreatic duct intervention was performed. Pancreatography via the punctured upstream pancreatic duct revealed a complete cut-off at the duct genu with failure to cannulate the duct downstream (**Figure 4B**). Thus, a 7-Fr, 7-cm double pigtail stent was placed across the fistula between the duct body and the stomach, creating pancreaticogastrostomy fistula. A rendezvous-assisted endoscopic retrograde pancreatography (ERP) via the matured fistula was performed 6 weeks later. A 10-Fr, 7-cm straight biliary stent was successfully inserted across the disrupted duct via the duodenal papilla. The patient made an uneventful recovery and was discharged from the hospital 5 days later. A follow-up CT 2 months later showed complete resolution of the pseudocyst and pleural effusions. Four months later, a follow-up ERP showed complete anatomical restoration of the pancreatic duct, and the duct stent was retrieved (**Figure 4C**). The patient was well and symptom-free for at least a further 10 months of follow up since successful placement of a stent in the duct. The total length of care at our institution was about 426 days. The episode of care was summarized in **Figure 5**. During this time, genetic testing was conducted, and we found a heterozygous missense substitution in *SPINK1*, c.101A>G (p. Asn34Ser), but otherwise negative for *PRSS1* variants. Tests for other possible genetic variations related to chronic pediatric pancreatitis such as in *CFTR*, *CTRC*, or the gene for calcium sensing receptor (*CASR*) are not available in our institution. Family history investigations revealed no history of pancreatic diseases.

## Discussion

**Figure 6:**
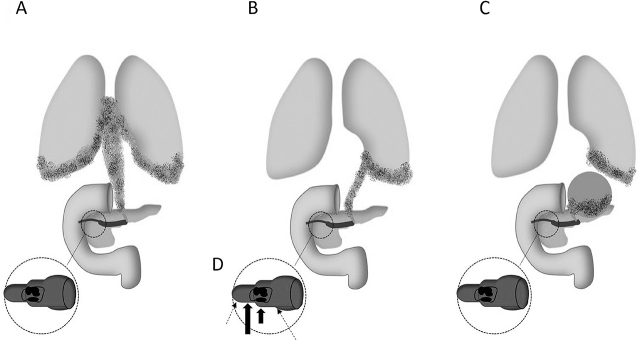
Patterns of a PPF (black–gray areas). (**A**) Mediastinal location: the pancreatic fluid leaks to the retroperitoneum and drains upwards to the mediastinum. Then the fluid leaks uni- or bilateral to one or both pleural cavities via hila or pleural fistula. (**B**) Diaphragmatic location: the pancreatic juice drains via the retroperitoneal space and erodes the diaphragm until the hiatus and to the pleural cavity. (**C**) A pseudocyst formed by anterior leakage from the pancreatic duct results in direct erosion of the diaphragm resulting in pleural effusion. (**D**) A magnified picture of the possible pathologies of the pancreatic duct in PPF: stricture of the focal downstream pancreatic duct (long arrow) causes dilatation of the upstream pancreatic duct (long dashed arrow) and pancreatic duct stone formation (short arrow). The undilated downstream pancreatic duct is also seen (short dashed arrow). PPF, pancreaticopleural fistula.

PPF is a rare cause of pleural effusion in children, mostly reported in the literature as case reports [1, 2, 8,9,10,11,12,13,14,15,16]. The pathophysiology of PPF involves the pancreatic fluid leaking directly from a focal pancreatic duct disruption or a pancreatic pseudocyst and moving upward into the thoracic cavity because of the pressure gradient between abdominal and thoracic cavities [18, 19]. This fluid can leak along the retroperitoneum, reaching toward the mediastinum or the diaphragm (**Figure 6A, B**) [18, 19]. Sometimes, the duct ruptures anteriorly and forms a pseudocyst in the lesser sac and the pancreatic juice then erodes the nearby diaphragm (**Figure 6C**) [19]. The causes of duct disruption can be direct trauma, or a late consequence of acute or chronic pancreatitis [2]. Focal pancreatic inflammation can result in a stricture, a protein plug, or stones in the duct, leading to increasing intraluminal pressure and disruption of the distal duct (**Figure 6D**) [18]. Diagnosis of PPF is established by the presence of an amylase-rich pleural effusion combined with direct visualization of the fistula, evidence of chronic pancreatitis or a pseudocyst in imaging [2, 20]. The diagnosis of PPF is often delayed because of the low incidence or lack of awareness of this abnormality [2, 20].

In addition to our case, we reviewed 12 other cases of PPF in children reported in the English literature 2001–2020 [1, 2, 8,9,10,11,12,13,14,15,161]. The patients’ ages ranged from 1.5 years to 13 years. All patients had presented with respiratory symptoms or vague abdominal pain with massive or intractable pleural effusion(s). The pleural effusion amylase content ranged from 950–157,000 IU/L. The details of the imaging findings in each case are shown in **Table 1**, and number of the abnormalities detected by imaging are provided in **Table 2** (n = 13). Most PPFs could be identified by extension to the mediastinum (6 cases) or diaphragm (5 cases). A pseudocyst without demonstrable fluid tract had been seen in only 1 case of the 13 (including ours) [8]. In our patient, MRCP was the most useful imaging to depict all pancreatic duct pathologies including PPF, duct disruption, irregular duct dilatation, proximal duct stricture, and stones. These findings by MRCP were made in 6, 3, 7, 2, and 2 of 7 other cases, respectively (**Table 2**). Nevertheless, CT provided the first imaging clues leading to diagnosis of PPF in our patient, namely evidence of chronic pancreatitis and a pseudocyst. Although the fistula was detected by CT in 7 of 12 cases, the tomography demonstrated some features of chronic pancreatitis including irregular dilated pancreatic duct in 8 cases, pancreatic calcifications in 4 cases, and pancreatic pseudocysts in 7 cases (**Table 2**). CT is more available and more practical for children than MRI, which usually requires sedation and pediatric-imaging specialized operators. Another advantage of CT is that when a child in whom PPF is suspected is scheduled for a chest CT to evaluate pulmonary conditions, the scan length can inferiorly extend to cover the pancreas. The initial information regarding PPF and/or chronic pancreatitis received by pancreatic CT will help determine whether the patient is able to proceed to specific treatments of PPF, or to undergo further investigation with MRCP. When compared to MRCP, ERCP had lower detection rate of the fistula, at 5 cases in 9, but produced better visualization of duct stricture and stones (**Table 2**). The diagnostic rate of PPF by ERCP was highly variable depending on the time of examination and anatomical variations [1, 2, 9, 10, 12, 15, 16]. The reported causes of failed ERCP in children with PPF included pancreas divisum, associated downstream duct stricture, and duct stone [1, 10, 12]. Ultrasound has only a small role in PPF, with detection rates for PPF of 0 in 6 cases, irregular dilatation of the duct in 3 cases, and pancreatic calcifications in 2 cases (**Table 2**).

**Table 1:** Imaging details of the reported cases of PPF in children including our case. Total patients (n = 13) [1, 2, 8,9,10,11,12,13,14,15,16].

**Case**	**Etiology**	**Imaging modalities**	**Modalities detecting fistula**	**Location of fistula**	**Location of pseudocyst**	**Direct disruption of PD**	**Proximal stricture of PD**	**PD stones**	**Irregular dilatation of PD (CP)**	**Other pancreatic findings**
Yang et al. [1]	CP	CT, MRCP, ERCP	MRCP	D	–	MRCP	–	ERCP	CT, MRCP	–
Zhang et al. [2]	CP	CT, MRCP, ERCP	MRCP, ERCP	D	Tail (CT, MRCP)	ERCP	ERCP	–	CT, MRCP	–
Gupta et al. [8]	AP	US, CT	–	–	Tail (US, CT)	–	–	–	–	AP-edema (CT)
Bishop et al. [9]	CP	MRCP, ERCP	ERCP	D	Head (MRCP)	ERCP	–	–	MRCP	–
Ranuh et al. [10]	CP	US, CT, ERCP	CT	M	–	CT	ERCP	–	–	CP-calcifications (US, CT)
Duncan et al. [11]	CP	US, CT	CT	M	Tail (US, CT)	–	–	–	US, CT	–
(2 cases)	CP	US, CT, IOC	CT, IOC	M	Head, tail (US, CT)	IOC	IOC	IOC	US, CT	CP-calcifications (US, CT)
Nacoti et al. [12]	CP	CT, MRCP, ERCP	CT, MRCP	M	Head (MRCP)	–	ERCP^†^	–	MRCP	Pancreas divisum (MRCP)
Ozbek et al. [13]	Trauma	US, CT	CT	D/M	Multiple (US, CT)	–	–	–	US, CT	–
Xiang and Zheng [14]	CP	US, CT	CT	M	Tail (US, CT)	–	–	–	–	–
Lee et al. [15]	CP	CT, MRCP, ERCP	MRCP	D	–	–	MRCP, ERCP	MRCP ERCP	CT, MRCP	–
Yu et al. [16]	CP	CT, MRCP, ERCP	MRCP, CT, ERCP	D	–	MRCP, CT, ERCP	–	ERCP	CT, MRCP	CP-calcifications (CT)
Present case	CP	CT, MRCP, ERCP	MRCP, ERCP	M	Body (CT, MRCP)	MRCP, ERCP	MRCP, ERCP	MRCP ERCP^‡^	CT, MRCP	–

–, not detected

† Stricture of Santorini's duct of pancreas divisum

‡ Stones in the pseudocyst.

CP, chronic pancreatitis; AP, acute pancreatitis; US, ultrasound; CT, computed tomography; MRCP, magnetic resonance cholangiopancreatography; ERCP, endoscopic retrograde cholangiopancreatography; IOC, intraoperative cholangiopancreatography; F, fistula; D, diaphragm; M, mediastinum; PD, pancreatic duct.

**Table 2:** Statistical summary of imaging findings of the reported cases of PPF in children including our case. Total patients (n = 13) [1, 2, 8,9,10,11,12,13,14,15,16].

**Features**	**Number of cases**
Site of pleural effusion (right: left: bilateral)	6: 4: 3
Number of USs	6
Number of CTs	12
Number of MRCPs	7
Number of ERCPs or IOCs	ERCP 8, IOC 1
Demonstrated fistulae	12/13
By US, CT, MRCP, ERCP or IOC	0/6, 7/12, 6/7, 5/9

**Type of fistula**	

Diaphragmatic	5
Mediastinal	6
Both diaphragmatic and mediastinal	1
Pseudocyst without direct fistula	1
PP detected by US, CT, MRCP	4/6, 7/12, 3/7
With fistulous tract by US, CT, MRCP	0/6, 4/12, 3/7
Without fistulous tract by US, CT, MRCP	4/6, 3/12, 0/7
Direct PD disruption	7/13
Detected by US, CT, MRCP, ERCP or IOC	0/6, 2/12, 3/7, 5/9
Proximal PD strictures	6/13
Visualized by US, CT, MRCP, ERCP or IOC	0/6, 0/12, 2/7, 6/9
PD stone	5/14
Visualized by US, CT, MRCP, ERCP or IOC	0/0, 0/12, 2/7, 5/9
PD irregular dilatation (CP)	11/13
Visualized by US, CT, MRCP	3/6, 8/12, 7/7
Pancreatic parenchymal calcifications by US, CT, MRCP	2/6, 4/12, 0/7
Pancreatic parenchymal edema by US, CT, MRCP	0/6, 1/12, 0/7
Pancreas divisum by MRCP, ERCP	1/13

US, ultrasound; CT, computed tomography; MRCP, magnetic resonance cholangiopancreatography; ERCP, endoscopic retrograde cholangiopancreatography; IOC, intraoperative cholangiopancreatography; PD, pancreatic duct; CP, chronic pancreatitis; PPF, pancreaticopleural fistula; PP, pancreatic pseudocyst.

Management of the fistula should be tailored on the morphologies of the pancreatic duct [2]. If the duct looks normal or is mildly dilated without stenosis, conservative treatment may be adequate. In cases of downstream ductal stenosis, partial ductal disruption in the pancreatic head or body, complete ductal disruption, ductal obstruction proximal to fistula, or impacted duct stones, endoscopic or surgical procedures should be considered [2].

**Figure 7:**
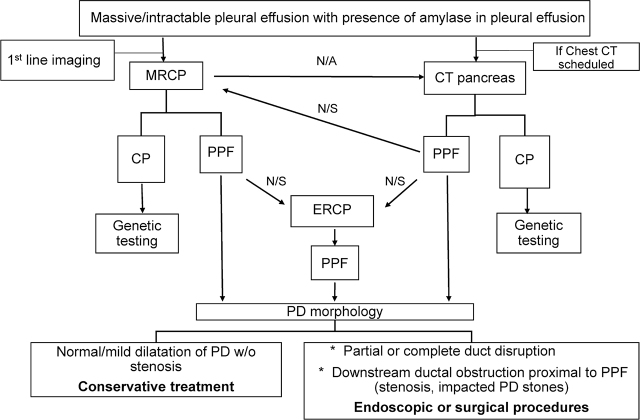
Flowchart of imaging and management guideline for cases of suspected PPF. MRCP, magnetic resonance cholangiopancreatography; CT, computed tomography; ERCP, endoscopic retrograde cholangiopancreatography; CP, chronic pancreatitis; PPF, pancreaticopleural fistula; PD, pancreatic duct; N/A, not available; N/S, not seen; w/o, without.

Chronic pancreatitis is the main etiology of PPF in children and genetic variations accounts for 73% of cases of chronic pancreatitis in children [2, 3]. One of the widely known pancreatitis-related variations is in *SPINK1* [3]. The *SPINK1* c.101A>G substitution variant, which is more frequently mentioned as the gene associated with chronic pancreatitis in patients with European ancestry than in patients with Asian ancestry, was, however, found in our Thai patient [3]. Other variants of *SPINK1* that have been previously reported are a c.194 + 2T>C substitution variant in a Chinese boy with PPF and a IVS3 + 2T>C slice-site substitution variant in many Chinese children with chronic pancreatitis [2, 3]. Limitations of this report include lack of some specific genetic tests related to chronic pancreatitis at Songklanagarind Hospital. Therefore, multifactorial genetic factors contributed to chronic pancreatitis could not be determined in our study. In summary, the imaging and management guidelines for suspected cases of PPF are shown in **Figure 7**.

## Conclusions

In children who present with massive pleural effusion with high levels of pleural fluid amylase, MRCP or pancreatic CT should be promptly conducted to enable the early diagnosis of PPF, which will reduce complications and shorten hospitalization in cases of pediatric PPF.
